# Inorganic Polyphosphate Is in the Surface of *Trypanosoma cruzi* but Is Not Significantly Secreted

**DOI:** 10.3390/pathogens13090776

**Published:** 2024-09-09

**Authors:** Logan P. Crowe, Anna Gioseffi, Mayara S. Bertolini, Roberto Docampo

**Affiliations:** 1 Center for Tropical and Emerging Global Diseases, University of Georgia, Athens, GA 30602, USAmaybertolini@uga.edu (M.S.B.); 2 Department of Cellular Biology, University of Georgia, Athens, GA 30602, USA

**Keywords:** cardiac fibrosis, Chagas disease, transforming growth factor beta, polyphosphate, *Trypanosoma cruzi*

## Abstract

*Trypanosoma cruzi* is the etiologic agent of Chagas disease, an infection that can lead to the development of cardiac fibrosis, which is characterized by the deposition of extracellular matrix (ECM) components in the interstitial region of the myocardium. The parasite itself can induce myofibroblast differentiation of cardiac fibroblast in vitro, leading to increased expression of ECM. Inorganic polyphosphate (polyP) is a linear polymer of orthophosphate that can also induce myofibroblast differentiation and deposition of ECM components and is highly abundant in *T. cruzi*. PolyP can modify proteins post-translationally by non-enzymatic polyphosphorylation of lysine residues of poly-acidic, serine-(S) and lysine (K)-rich (PASK) motifs. In this work, we used a bioinformatics screen and identified the presence of PASK domains in several surface proteins of *T. cruzi*. We also detected polyP in the external surface of its different life cycle stages and confirmed the stimulation of host cell fibrosis by trypomastigote infection. However, we were not able to detect significant secretion of the polymer or activation of transforming growth factor beta (TGF-β), an important factor for the generation of fibrosis by inorganic polyP- or trypomastigote-conditioned medium.

## 1. Introduction

Chagas disease is the most prevalent parasitic disease in the Americas, affecting 6 to 7 million people, with 100 million people at risk worldwide [1,2]. In the U.S., it is estimated that 300,000 individuals are infected and unknowingly expose others to infection through blood and tissue donation [3]. No vaccines are available against Chagas disease, despite recent efforts [4,5,6], and several challenges remain with the current anti-parasitic drug treatment including partial or lack of activity in the acute or chronic stage of the disease, respectively, and unwanted side effects [7,8]. Chagas cardiopathy is the most frequent clinical presentation of the chronic disease, which develops in about 30% of the infected patients [9]. Acute myocarditis can occur in about 1% of infected patients [10]. Interstitial fibrosis occurs in both acute and chronic heart disease and is characterized by deposition of extracellular matrix (ECM) components by cardiac myofibroblasts in the interstitial region of the myocardium [10]. The mechanism and the molecular signaling pathways responsible for the deposition of ECM resulting from the infection with *T. cruzi* are not known, although transforming growth factor beta (TGF-β) was proposed to mediate cardiac fibrosis [11].

Fibrosis results from excessive accumulation of ECM by terminally differentiated fibroblasts (myofibroblasts) in response to injury or illness and leads to organ disfunction and failure. Myofibroblasts are the main cellular effectors in cardiac fibrosis and the main source of matrix proteins [12]. The most abundant ECM components are the collagens, elastins, fibronectins, and proteoglycans [13]. TGF-β is the best-characterized fibrogenic growth factor [14]. It is secreted by many cell types in a latent form and needs to be activated through proteolysis or interaction with ECM proteins to perform its biological role [15]. Once active, TGF-β binds to membrane receptors (type I and type II) and stimulates phosphorylation of proteins via the Smad2/3 proteins or via alternative pathways, MAPK, JNK, p38, and PI3K, inducing a variety of cellular responses including the regulation of more than 500 genes [15]. TGF-β induction and activation has been demonstrated in several models of cardiac fibrosis and in human hearts with fibrotic cardiomyopathic changes [16]. Chagas cardiac fibrosis was proposed to be mediated by TGF-β, as shown by its involvement in the production of several ECM components [11].

Acute Chagas myocarditis’ residual effects include interstitial fibrosis and myofiber hypertrophy, with minimal inflammation, while the chronic phase of Chagas cardiomyopathy includes low-grade myocarditis with myofiber hypertrophy and interstitial fibrosis that develops over years to decades [10]. Deposition of ECM components (fibronectin, laminin, and collagen) in the infected heart was demonstrated in three-dimensional cardiomyocyte cultures [17] and in cardiac myofibroblasts [18] infected with the parasite. *T. cruzi* was possibly responsible as no inflammatory cells were present, and treatment with posaconazole reverted this effect [19].

Suess et al. [20] demonstrated that polyphosphate (polyP) is a potent inducer of fibroblast chemotaxis, myofibroblast differentiation, and production of ECM components, such as a-smooth muscle actin (α-SMA), stress fibers, and collagen. PolyP is a polymer of three to hundreds of orthophosphate units bound by high-energy phosphoanhydride bonds and is highly abundant in *T. cruzi* [21]. We have found that polyP is released by cells such as platelets [22], and mast cells [23]. Platelet-released polyP is a potent procoagulant [24] and proinflammatory [25] agent, and acts as a chemotactic agent for neutrophils, and a stimulant of fibroblast differentiation to myofibroblasts [20]. PolyP present in extracellular vesicles derived from prostate cancer cells (prostasomes) activates coagulation Factor XII [26]. PolyP is also present in extracellular vesicles from different cancer cells and mediates binding of Factor XII, contact activation, and thrombosis [27].

PolyP has been implicated in many processes from serving as an energy source to regulating gene expression [28,29]. In *T. cruzi*, polyP was first found in acidocalcisomes [21]. However, using a combination of biotinylated polyP and a bioinformatics approach, several glycolytic/gluconeogenic proteins, ribosomal proteins, and other nucleolar proteins that putatively interact with this polymer were identified, and polyP was detected in the glycosomes and nucleoli of these parasites using the polyP-binding domain of *E. coli* exopolyphosphatase (PPBD) coupled to Alexa 488 [30].

Azevedo et al. [31] first reported that polyP can be attached to lysine residues of proteins within PASK domains and used the term polyphosphorylation to define this non-enzymatic post-translational modification. This modification was found initially in two yeast proteins, nuclear signal recognition 1 (Nsr1), and its interacting partner topoisomerase 1 (Top1). Polyphosphorylation downregulated their interaction and inhibited Top1 enzymatic activity [31]. Further work found evidence that polyphosphorylation can occur in several other *Saccharomyces cerevisiae* [32,33,34], as well as in human [32] enzymes.

In this work, we used a bioinformatics screen developed by Bentley-DeSousa et al. [32] to search the proteome of *T. cruzi* for proteins harboring putative PASK-like domains. Interestingly, we found many surface proteins containing this domain and that polyP was present on the surface of *T. cruzi* at different life stages. Since it has been reported that a trypomastigote invasion of host cells leads to fibrosis and polyP has been shown to induce fibrosis of mammalian cells, we investigated whether polyP was released from the trypomastigote surface and stimulated fibrosis of host cells through TGF-β activation.

## 2. Materials and Methods

### 2.1. Chemicals and Reagents 

BCA Protein Assay Kit was from Thermo Fisher Scientific Inc. (Waltham, MA, USA) PureView prestained protein ladder was from Azura Genomics (Raynham, MA, USA). Alexa Fluor-conjugated secondary antibodies were purchased from Life Technologies (Carlsbad, CA, USA). Fluoromount-G^®^ was from SouthernBiotech (Birmingham, AL, USA). Nitrocellulose membranes were from Bio-Rad (Hercules, CA, USA). Recombinant human latent TGF-β 1 protein was from R&D Systems (Minneapolis, MN, USA). PolyP_60_ was a gift from Dr. Toshikazu Shiba (Kitasato University). All other reagents of analytical grade were from Sigma (St. Louis, MO, USA).

### 2.2. Cell Cultures 

*T. cruzi* Y strain epimastigotes were cultured in liver infusion tryptose (LIT) medium containing 10% heat-inactivated newborn calf serum at 28 °C [35]. Tissue culture cell-derived trypomastigotes [36] and amastigotes [37] were obtained from infected Vero cells and were collected from the culture medium of infected host cells as described in the references. Vero cells were grown in RPMI supplemented with 10% fetal bovine serum and maintained at 37 °C with 5% CO_2_.

### 2.3. PASK Domain Screen

The proteomes of *T. cruzi*, *T. brucei*, and *L. major* were screened for putative PASK domains using a custom script designed by Bentley-Souza et al. [32]. In short, parasite proteomes were retrieved from Uniprot database and searched for proteins containing at least 20 amino acid clusters of ≥75% S/E/D/K with at least one K residue. The identified proteins were analyzed for gene ontology (GO) term enrichment using TritrypDB (VEuPathDB.org accessed on 9 May 2024).

### 2.4. Polyphosphate Surface Staining

Detection of polyphosphate on the cell surface was performed using the polyP-binding domain (PPBD) from *E. coli* PPX [38] conjugated with Alexa Fluor 488 (Molecular Probes, Eugene, OR, USA) as described before [30]. Cells were harvested, washed 2× with PBS, and fixed for 15 min with 4% paraformaldehyde in 1× PBS pH 7.4. Cells were allowed to settle on poly-L-lysine-coated cover slips for 30 min and blocked with 5% goat serum, 3% BSA, and 1% fish gelatin for 1 h, at RT. The coverslips were incubated with 8 μg/mL PPBD-Alexa fluor 488 and 5 μg/mL DAPI for 1 h, at RT in the dark. Following incubation, the coverslips were washed 3× with PBS and mounted on slides using fluoromount G. The slides were imaged on a Deltavision deconvolution microscope (GE) equipped with a 100× objective NA 1.35.

### 2.5. In Vitro Fibroblast Infection Assay

NIH-3T3 cells cultured in DMEM containing high glucose, pyruvate (Thermo Scientific 11995065, Boston, MA, USA), 1% penicillin and streptomycin, 10% heat-inactivated newborn calf serum, and 10 mM Hepes were seeded at 5 × 10^4^ per well in a 6-well dish with glass coverslips, then allowed to incubate for 24 h. Fibroblasts were then rinsed with Dulbecco’s Hanks’ solution, and tissue culture-derived *T. cruzi* trypomastigotes (Y strain) were used to infect at a 20:1 ratio of parasites to host cells. At 8 h post-infection, coverslips were washed with Dulbecco’s Hanks’ solution to remove extracellular parasites, and the media was replaced. At 24, 48, and 72 h post initiation of infection, coverslips were removed from wells and immediately fixed in 4% paraformaldehyde in PBS, pH 7.4, at room temperature for 15 min. Coverslips were washed once with PBS pH 7.4, then stored in PBS at 4 °C prior to immunofluorescence assay. Uninfected controls were treated in the same manner, without the addition of parasites.

### 2.6. Immunofluorescence Analyses

To evaluate fibroblast differentiation as evidenced by relative levels of α-SMA, fixed coverslips were permeabilized for 5 min with 0.1% Triton X-100. The cells were then blocked overnight at 4 °C with PBS containing 50 mM NH_4_Cl, 3% BSA, 5% goat serum, and 1% fish gelatin. Next, the coverslips were incubated with 1:200 monoclonal rabbit anti-α-SMA antibody (ABclonal A17910; Woburn, MA, USA) diluted in 1% BSA in PBS, pH 8.0, for 1 h at room temperature. The coverslips were washed three times with 1% BSA in PBS, pH 8.0, then incubated with 1:1000 TRITC-conjugated goat-anti-rabbit antibodies for 1 h in the dark at room temperature. The coverslips were then washed twice with 1% BSA in PBS, pH 8.0, followed by a final wash with PBS, pH 8.0. Then, the coverslips were mounted on slides using Fluoromount-G mounting medium containing 5 μg/mL of DAPI to stain DNA. The fluorescence images were captured using the DeltaVision II microscope system (Applied Precision, Inc., Issaquah, WA, USA), with a 60× objective using equivalent parameters for each image capture. The images were converted to gray scale and opened in FIJI. Using a pre-assigned circular ROI of equal size and a mean gray area of the TRITC channel for one background region, three randomly chosen regions per cell in frame were measured. Five images were analyzed for each coverslip, with three biological replicas. The mean gray area of the background was subtracted from the mean gray area of cells for each image, giving a normalized measurement of fluorescence in relative quantification units. Data were then compiled, graphed, and analyzed using GraphPad Prism software version 9 (GraphPad, La Jolla, CA, USA). The reported values are means ± standard deviation from 3 biological experiments. The level of significance was evaluated by one-way analysis of variance (ANOVA) with Tukey’s multiple comparisons test.

### 2.7. Secretion and Extracellular Vesicle Isolation

Cell culture-derived trypomastigotes were collected, washed, and resuspended at 1 × 10^8^ parasites per milliliter in 10 mL of buffer A with glucose, pH 5 (BAG; 116 mM NaCl, 5.4 mM KCl, 0.8 mM MgSO_4_, 50 mM Hepes, 5.5 mM glucose). Parasites were then incubated for 24 h at 37 °C and 5% CO_2_ to allow for secretion of molecules and release of extracellular vesicles. At the endpoint of incubation, parasites were resuspended homogenously and counted to determine the terminal cell counts. The total conditioned media and parasites were collected and centrifuged at 3000× *g* for 10 min to pellet cells. After collecting the supernatant for the next step, the cell pellet was washed with fresh, sterile BAG, pH 7.0, then rapidly frozen using liquid nitrogen and stored at −80 °C. The supernatant was then passed through a 0.45 μm syringe filter to remove any remaining cellular debris. An aliquot of the conditioned media filtrate was then rapidly frozen and stored at −80 °C to represent “total secretion”. The remaining filtrate was then centrifuged at 100,000× *g* and 4 °C for 2 h to pellet large extracellular vesicles (V2) followed by a 16 h spin to pellet smaller extracellular vesicles (V16) [39]. Vesicle pellets were resuspended in 50 μL of fresh, sterile BAG, pH 7.0, then rapidly frozen in liquid nitrogen and stored at −80 °C. An aliquot of the final EV-depleted supernatant was then collected, rapidly frozen, and stored at −80 °C. Extraction and measurement of short chain polyP was conducted as previously reported [21]. Briefly, samples were resuspended in 1 M perchloric acid and sonicated on ice. Cellular debris were pelleted by centrifuging at 18,000× *g* for 5 min at 4 °C. The cleared supernatant was then added to 4 mg of washed TiO_2_ beads and allowed to incubate for 20 min at 4 °C with rotation. The TiO_2_ beads were then washed twice with 1 M perchloric acid and pelleted. Bound polyphosphates were then eluted by addition of 400 µL total of cold 2.8% ammonium hydroxide in two parts. Sample volumes were reduced using a centrifugal evaporator until the pH of the sample was 7 to 8. Extracted polyP samples were then rapidly frozen and stored at −80 °C for analysis.

### 2.8. TGF-β Activation

Latent TGF-β 1 (10 ng/mL) was incubated with a DMEM medium containing 1.5% bovine calf serum or with a trypomastigote-conditioned medium in the presence or absence of plasmin (2 U/mL) and in the presence or absence of 5 µM polyP_60_ (a gift from Toshikazu Shiba, Kitasato University) for 2 h at 37 °C. To obtain the conditioned media from trypomastigotes, the cell-derived trypomastigotes were incubated in phosphate-free buffer A with glucose (BAG, 116 mM NaCl, 5.4 mM KCl, 0.8 mM MgSO_4_, 50 mM Hepes pH 7.4, and 5.5-mM glucose), at a concentration of 1 × 10^8^ parasites per ml at 37 °C in 5% CO_2_ for 24 h. This conditioned medium was clarified by centrifugation at 2000× *g* for 10 min and filtered through a 0.45 µm membrane.

### 2.9. Western Blot Analyses

The samples were mixed with a 4× Laemmli sample buffer (125 mM Tris-HCl, pH 7, 20% (*v*/*v*) glycerol, 4.0% (*w*/*v*) SDS, 4.0% (*w*/*v*) bromophenol blue) before application to 10% SDS-polyacrylamide gels. The separated proteins were transferred onto nitrocellulose membranes with a Bio-Rad Trans-blot apparatus. The membranes were blocked with 5% nonfat dried skim milk in PBS-T (PBS containing 0.1% *v*/*v* Tween 20) overnight at 4 °C. Next, the membranes were incubated for 1 h at room temperature with the primary antibody TGF beta 1 Rabbit from ABclonal (1:2000). After three washes with PBS-T, the blots were incubated with the secondary antibody IRDye 680RD-conjugated goat anti-rabbit IgG (1:10,000) for 1 h at room temperature in the dark. The blots were washed three times with PBS-T, and the Western blot images were obtained and processed with the Odyssey infrared imaging system (LI-COR Biosciences, Lincoln, NE, USA). The densities of the Western blot bands were quantified with the ImageStudioLite software v.6 (LI-COR Biosciences).

## 3. Results

### 3.1. Bioinformatics Screen of Trypanosomatid Proteins

**Figure 1:**
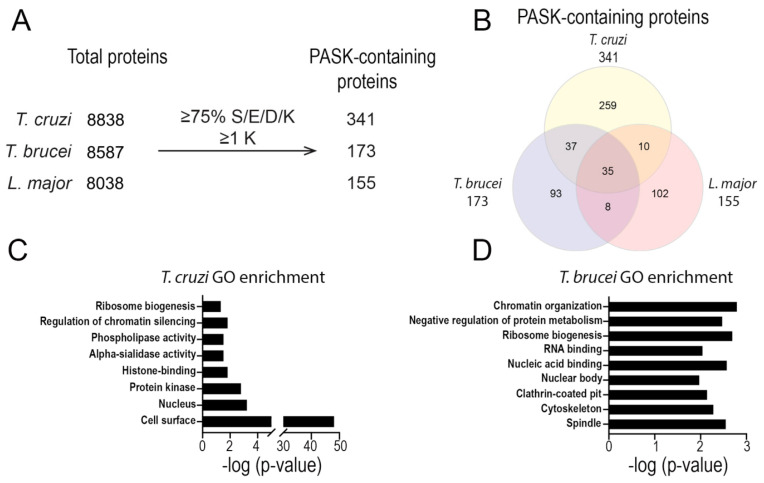
**Proteins with a PASK domain in trypanosomatids**. (**A**,**B**) Number of proteins identified as possessing a PASK domain in the proteomes of *T. cruzi*, *T. brucei*, and *L. major*. (**C**,**D**) Analysis of the proteins from *T. cruzi* (**C**) or *T. brucei* (**D**) according to molecular function. GO terms found for proteins possessing a putative PASK domain are indicated.

Using a bioinformatics screen developed before [32], we searched the proteomes of *T. cruzi*, *T. brucei*, and *L. major* for proteins possessing putative PASK domains. In total, we found 341 *T. cruzi* proteins, 173 *T. brucei* proteins, and 155 *L. major* proteins that possess regions of 20 amino acid residues containing at least 75% serine (S)/glutamic acid (E)/aspartic acid (D)/lysine (K) residues with at least 1 lysine (Figure 1A and Table S1). Of these proteins, 35 have orthologs present in each kinetoplastid parasite species (Figure 1B). To determine the function of these putative PASK domain-containing proteins, we performed gene ontology (GO) term analysis using TriTrypDB.org. We observed enrichment for proteins involved in processes such as ribosome biogenesis, histone binding, RNA binding, and chromatin organization in both *T. cruzi* and *T. brucei* (Figure 1C,D). Unexpectedly, we also saw strong enrichment for proteins annotated to be in the cell surface, especially in *T. cruzi* (Figure 1C). In total, 9 *trans*-sialidases, 2 TcMUC II, and 103 mucin-associated surface proteins [MASP] were found to have a PASK domain (Table S1).

### 3.2. Polyphosphate Localization in the Outer Surface of T. cruzi

**Figure 2:**
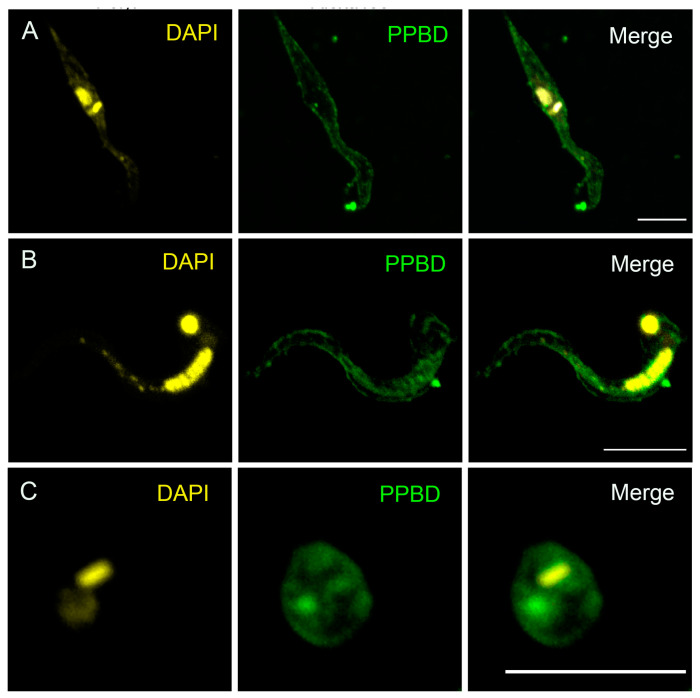
**Presence of surface polyP in *T. cruzi* different stages.** (**A**) Fluorescence analysis of non-permeabilized *T. cruzi* wild-type epimastigotes stained with DAPI (left) or PPBD-Alexa488 (middle) and merged images (right) showing the K-DNA and nuclei in *yellow* and surface expression of polyP in *green*, respectively. Scale bar = 5 µm. (**B**,**C**) Fluorescence analysis of non-permeabilized trypomastigotes (**B**) and amastigotes (**C**) stained with DAPI (left), and PPBD-Alexa 488 (PPBD) (middle) and merged images (right). Note that DAPI is cell permeable and strongly labels the nucleus, and k-DNA. Scale bars = 5 µm.

In previous work, we used Alexa Fluor 488-labeled polyP-binding domain (PPBD) of *E. coli* exopolyphosphatase to investigate the presence of polyP in glycosomes and nucleoli of permeabilized *T. cruzi* and *T. brucei* different stages [30]. Because we found that several outer-surface proteins of *T. cruzi* have PASK domains (Figure 1), we used Alexa fluor 488-labeled PPBD in non-permeabilized trypanosomes, and we detected labeling of polyP at the outer surface of the plasma membrane of epimastigotes (Figure 2A), a localization that has also been reported in fungi [40]. DAPI, which is permeable, labeled the kinetoplast and nuclear DNA. Importantly, we also found surface membrane expression of polyP in *T. cruzi* trypomastigotes (Figure 2B), and amastigotes (Figure 2C), the infective stages. No surface labeling was found in the permeabilized cells, as reported before [30].

### 3.3. T. cruzi Infection Induces Myofibroblast Differentiation in NIH-3T3 Cells

**Figure 3:**
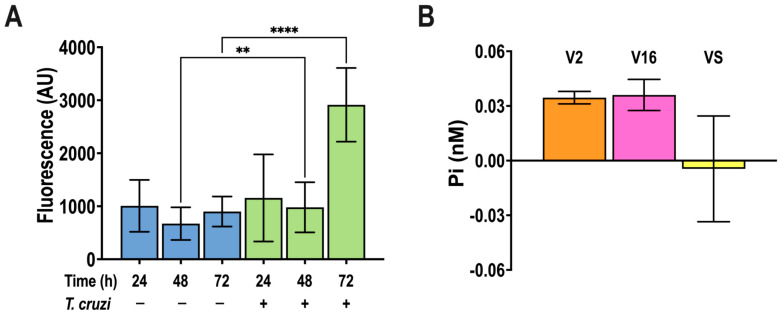
**α-SMA production by *T. cruzi*-infected NIH-3T3 cells, and polyP presence in trypomastigote extracellular vesicles.** (**A**) NIH-3T3 cells were infected by *T. cruzi* cell-derived trypomastigotes (ratio 20:1) and α-SMA antibody (1:200) staining was quantified by fluorescence microscopy as a marker of myofibroblast differentiation at different times post-infection. Values are means ± S.D., *n* = 3, ** *p* < 0.01, **** *p* < 0.0001, ANOVA with Turkey’s multiple comparisons test. (**B**) PolyP extracted from large (V2) and small (V16) extracellular vesicles, and vesicle free supernatant (VS) of 1 × 10^8^/mL cell-derived trypomastigotes incubated for 18 h, at pH = 5.0, detected by the PPX method, and expressed in P_i_ units.

α-Smooth muscle actin (α-SMA) is a cytoskeletal protein that is highly expressed in activated myofibroblasts [41]. By assessing the changes in α-SMA expression, it has been shown that *T. cruzi* infection activates the differentiation of cardiac fibroblast into myofibroblasts [18]. In agreement with those results, the infection of NIH-3T3 fibroblasts with *T. cruzi* trypomastigotes showed a time-dependent increase in α-SMA expression, as detected by immunofluorescence analysis using specific antibodies (Figure 3A).

### 3.4. Presence of PolyP in Extracellular Vesicles of T. cruzi

*T. cruzi* trypomastigotes have been shown to release proteins associated with vesicles obtained by fractionation of conditioned cultured supernatant [39]. We applied this fractionation technique and measured their polyP content, as described in Materials and Methods but found only nanomolar levels of polyP (expressed as P_i_ units) in large (V2) and small (V16) extracellular vesicles and negligible levels in the vesicle supernatant (VS) (Figure 3B).

### 3.5. Lack of Activation of TGF-β by PolyP- or T. cruzi Trypomastigotes-Conditioned Medium

**Figure 4:**
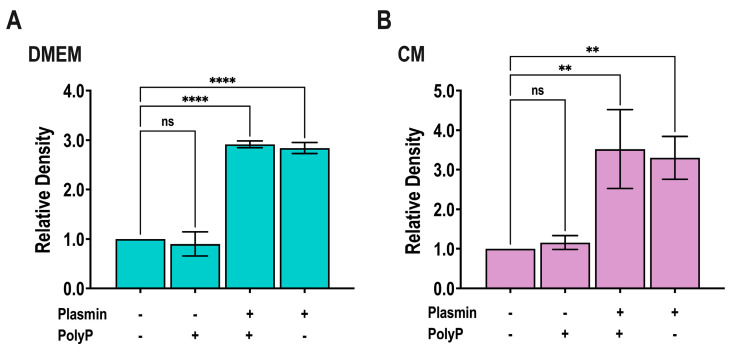
**Activation of latent TGF-β by plasmin and conditioned medium with and without polyP_60_.** (**A**,**B**) Latent TGF-β was incubated with or without plasmin (2 U/mL) in DMEM with 1.5% bovine calf serum (**A**) or in conditioned medium (**B**) for 2 h at 37 °C, in the absence or presence of 5 µM polyP_60_. Samples were prepared for PAGE analysis followed by immunoblot analysis to detect the 13 kDa mature TGF-β, and band density was measured. Values are means ± SD (n = 3). ** *p* < 0.01 and **** *p* < 0.0001; n.s., no significant, by one-way ANOVA with Dunnett’s multiple comparison test.

We investigated whether TGF-β is activated by polyP- or *T. cruzi* trypomastigotes conditioned medium. Plasmin, a known TGF-β activator [42], was used as positive control. Briefly, latent TGF-β was incubated with plasmin (2 U/mL) in a DMEM- or *T. cruzi*-conditioned medium for 2 h at 37 °C, in the absence or presence of 5 µM polyP_60_ (the polyP size and conditions used in [20] to demonstrate fibrosis stimulation by polyP). Triplicate samples were prepared for PAGE followed by immunoblot analysis to detect the 13 kDa mature TGF-β. While plasmin was able to activate TGF-β, conditioned medium with or without polyP was not able to activate it and polyP did not potentiate the plasmin effect (Figure 4 and Figure S1).

## 4. Discussion

The use of the polyP-binding domain from *E. coli* PPX conjugated with Alexa Fluor 488 allowed us to demonstrate the presence of polyP on the external surface of *T. cruzi* different life cycle stages. These results are in agreement with the presence of PASK domain-containing proteins in several surface proteins of the parasite, suggesting that these proteins could be polyphosphorylated. We confirmed the ability of *T. cruzi* infective forms to induce myofibroblast differentiation, but we found little evidence that this was caused by secreted polyP or that a trypomastigote-conditioned media were able to induce activation of the fibrosis-inducing TGF-β. However, we cannot rule out the possibility that bound polyP is involved in inducing myofibroblast differentiation.

Our bioinformatics screen of PASK domain-containing proteins identified a lower number of proteins in trypanosomatids than in yeast, but gene ontology-term analysis indicated enrichment in similar functions related to ribosome biogenesis and rRNA processing and proteins with nuclear and nucleolar localizations. Unexpectedly, several of the identified proteins have an external surface localization (mucin-associated surface protein (MASP), *trans*-sialidase, mucin TcMUCII, and phosphoinositide phospholipase C (PI-PLC) in *T. cruzi*; variant surface glycoprotein (VSG) in *T. brucei*, and amastin-like proteins in *L. major*) (Table S1). Accordingly, polyP was localized to the external surface of *T. cruzi* different life cycle stages, and it is tempting to speculate that it could be attached to these surface proteins by polyphosphorylation. Further work will be required to demonstrate that this is the case. PolyP has been linked to the persistence of *T. cruzi* infection [43] and it is possible that this surface localization could contribute to this property.

Until now, only a few proteins have been reported as susceptible to polyphosphorylation in yeast and humans. After the initial description of polyphosphorylation of Nsr1 and Top1 [31], the use of yeast in the bioinformatics screen that we used here led to the selection of a group of 90 potential targets, of which 23 were confirmed as susceptible to polyphosphorylation by the protein mobility shift assay [32,34]. A first group of 15 protein targets included several proteins implicated in ribosome biogenesis [32] while a second group of 8 proteins were shown to interact with previous targets implicated in ribosome biogenesis or those that consisted of vacuolar proteins [34]. Six human proteins were found to be polyphosphorylated following expression of *Escherichia coli* polyP kinase (PPK) in HEK293T cells, some of them homologs to the yeast targets [32]. Screening of a human protein array with biotinylated polyP identified eight additional human proteins susceptible to polyphosphorylation [33]. Early work showed that polyphosphorylation could affect protein–protein interaction, subcellular localization, or enzymatic activity of the targeted proteins [31].

*T. cruzi*, like many other cells, has been demonstrated to release extracellular vesicles [39]. The surface localization of polyP suggested that this polymer could possibly be detected in these vesicles and be involved in inducing myofibroblast differentiation and deposition of ECM components. However, only nanomolar levels of polyP could be detected in small and large extracellular vesicles released by infective trypomastigotes while micromolar amounts of polyP were shown to be needed to stimulate ECM components deposition in NIH-3T3 fibroblasts [20]. Neither the *T. cruzi* trypomastigote-conditioned medium nor synthetic polyP were able to induce TGF-β activation, a reported mediator of cardiac fibrosis [14].

In summary, our work identified the surface localization of polyP and confirmed the myofibroblast deposition of ECM components upon *T. cruzi* infection of mammalian cells, but did not find significant secretion of polyP or stimulation of TGF-β activation by a polyP- or trypomastigote-conditioned medium.

## Data Availability

Data are contained within the article and Supplementary Information.

## Supplementary Materials

The following supporting information can be downloaded at: https://www.mdpi.com/article/10.3390/pathogens13090776/s1, Figure S1: Latent TGF-β activation; Table S1: *T. cruzi*, *T. brucei*, and *L. major* proteins possessing putative PASK domains.
