# Supplementary material for: Inorganic Polyphosphate Is in the Surface of Trypanosoma cruzi but Is Not Significantly Secreted
Source: Pathogens. 2024 Sep 9;13(9):776. doi: 10.3390/pathogens13090776 (PMC11434814; doi:10.3390/pathogens13090776)
Supplement: Supplementary file 1 [file pathogens-13-00776-s001.zip › pathogens-3174496-R1-supplementary.pdf]

Supplementary Information

Table S1. *T. cruzi*, *T. brucei*, and *L. major* proteins possessing putative PASK domains.

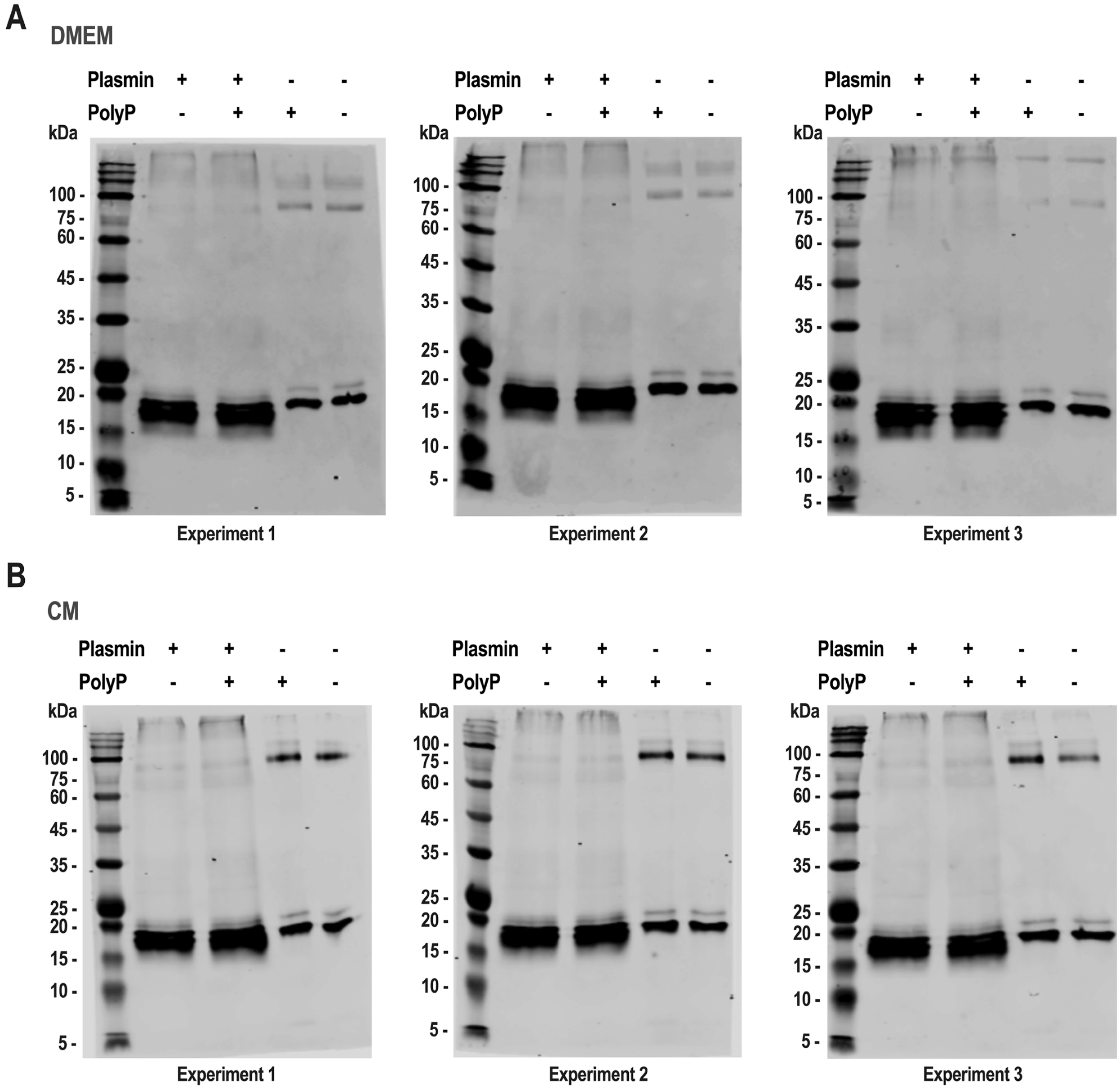

Figure S1. Latent TGF-β activation. **A, B,** Latent TG F-β was incubated with or without plasmin (2 U/ml) in DMEM with 1.5% bovine calf serum (**A**) or in conditioned medium (**B**) for 2 h at 37°C, in the absence or presence of 5 μM $polyP_{60}$. Immunoblot analysis detected the 13-kD mature TGF-β.
